# Amyloid-β Oligomers Regulate ADAM10 Synaptic Localization Through Aberrant Plasticity Phenomena

**DOI:** 10.1007/s12035-019-1583-5

**Published:** 2019-04-13

**Authors:** Elena Marcello, Stefano Musardo, Lina Vandermeulen, Silvia Pelucchi, Fabrizio Gardoni, Nadia Santo, Flavia Antonucci, Monica Di Luca

**Affiliations:** 1 Department of Pharmacological and Biomolecular Sciences, Università degli Studi di Milano, Via Balzaretti 9, 20133 Milan, Italy; 2 Present Address: Department of Basic Neuroscience, University of Geneva, Rue Michel-Servet 1, 1206 Geneva, Switzerland; 3 Department of Neurosciences, Psychology, Drug Research, and Child Health, University of Florence, Florence, Italy; 4 Department of Life Sciences, Università degli Studi di Milano, Milan, Italy; 5 Department of Biotechnology and Translational Medicine, Università degli Studi di Milano, Milan, Italy

**Keywords:** ADAM10, Alzheimer disease, Amyloid-β, Synaptic plasticity

## Abstract

A disintegrin and metalloproteinase 10 (ADAM10) is a synaptic enzyme that has been previously shown to limit amyloid-β_1–42_ (Aβ_1–42_) peptide formation in Alzheimer’s disease (AD). Furthermore, ADAM10 participates to spine shaping through the cleavage of adhesion molecules and its activity is under the control of synaptic plasticity events. In particular, long-term depression (LTD) promotes ADAM10 synaptic localization triggering its forward trafficking to the synapse, while long-term potentiation elicits ADAM10 internalization. Here, we show that a short-term in vitro exposure to Aβ_1–42_ oligomers, at a concentration capable of inducing synaptic depression and spine loss, triggers an increase in ADAM10 synaptic localization in hippocampal neuronal cultures. However, the Aβ_1–42_ oligomers-induced synaptic depression does not foster ADAM10 delivery to the synapse, as the physiological LTD, but impairs ADAM10 endocytosis. Moreover, Aβ_1–42_ oligomers-induced inhibition of ADAM10 internalization requires neuronal activity and the activation of the NMDA receptors. These data suggest that, at the synaptic level, Aβ_1–42_ oligomers trigger an aberrant plasticity mechanism according to which Aβ_1–42_ oligomers can downregulate Aβ generation through the modulation of ADAM10 synaptic availability. Moreover, the increased activity of ADAM10 towards its synaptic substrates could also affect the structural plasticity phenomena. Overall, these data shed new lights on the strict and complex relationship existing between synaptic activity and the primary mechanisms of AD pathogenesis.

**Electronic supplementary material:**

The online version of this article (10.1007/s12035-019-1583-5) contains supplementary material, which is available to authorized users.

## Background

Disentangling the initial steps of Alzheimer’s disease (AD) pathogenesis from full-blown pathology at a molecular and cellular level remains a key step to fully understand disease onset and progression. In this frame, it has been shown that synapse dysfunction and spine loss represent an early event of the disease rather than just a consequence of cell death [1]. Further, the synapses have been shown to be the main target of the amyloid-β_1–42_ (Aβ_1–42_) peptide, whose deposition is one of the main hallmarks of AD [2, 3].

The Aβ_1–42_ peptide derives from a transmembrane protein, named amyloid-β precursor protein (APP), that is mainly localized in the pre-synaptic active zone and in the post-synaptic compartment in the hippocampus and in the cortex [4].

The concerted action of the β-secretase BACE-1 and the γ-secretase towards APP generates Aβ_1–42_ [5]. Being APP cleavage mutually exclusive, in neuronal cells, A disintegrin and metalloproteinase 10 (ADAM10) cleaves APP within the Aβ_1–42_ domain, thus generating the neuroprotective sAPPα and precluding the formation of the Aβ_1–42_ peptide [6, 7].

The Aβ_1–42_ homeostasis is regulated by synapse activation: increased activity enhances secretion of Aβ_1–42_, while reduced activity inhibits it [8, 9]. Coherently, also ADAM10 synaptic levels and activity towards APP are under the control of activity-dependent synaptic plasticity [10]. Long-term depression (LTD) boosts ADAM10 membrane insertion by fostering its SAP97-mediated forward trafficking to post-synaptic membrane, whereas long-term potentiation (LTP) reduces the enzyme membrane levels by inducing AP2-dependent endocytosis [10].

On the other hand, Aβ_1–42_ can be considered a regulator of neuronal activity [8] since once released, it affects in turn synaptic transmission and plasticity. In particular, pathological Aβ_1–42_ levels giving rise to the formation of Aβ_1–42_ oligomers (oAβ_1–42_) may indirectly cause a partial block of N-methyl-D-aspartate (NMDA)-type glutamate receptors and shift the activation of NMDA receptors-dependent signaling cascades towards pathways involved in the induction of LTD and synaptic loss [11–13].

Here, we hypothesize that the oAβ_1–42_-induced plasticity pathways have a feedback effect on ADAM10 synaptic localization. We show that short-term exposure to oAβ_1–42_ reduces ADAM10 endocytosis, thus leading to an increase in ADAM10 synaptic localization. This effect is mediated by activation of synaptic NMDA receptors containing the GluN2A subunit.

## Results

### Characterization of oAβ_1–42_ Effect on the Synapse

In order to set up a reliable in vitro system to analyze the effect of oAβ_1–42_ on ADAM10 synaptic localization, we performed a complete characterization of our experimental conditions.

**Fig. 1:**
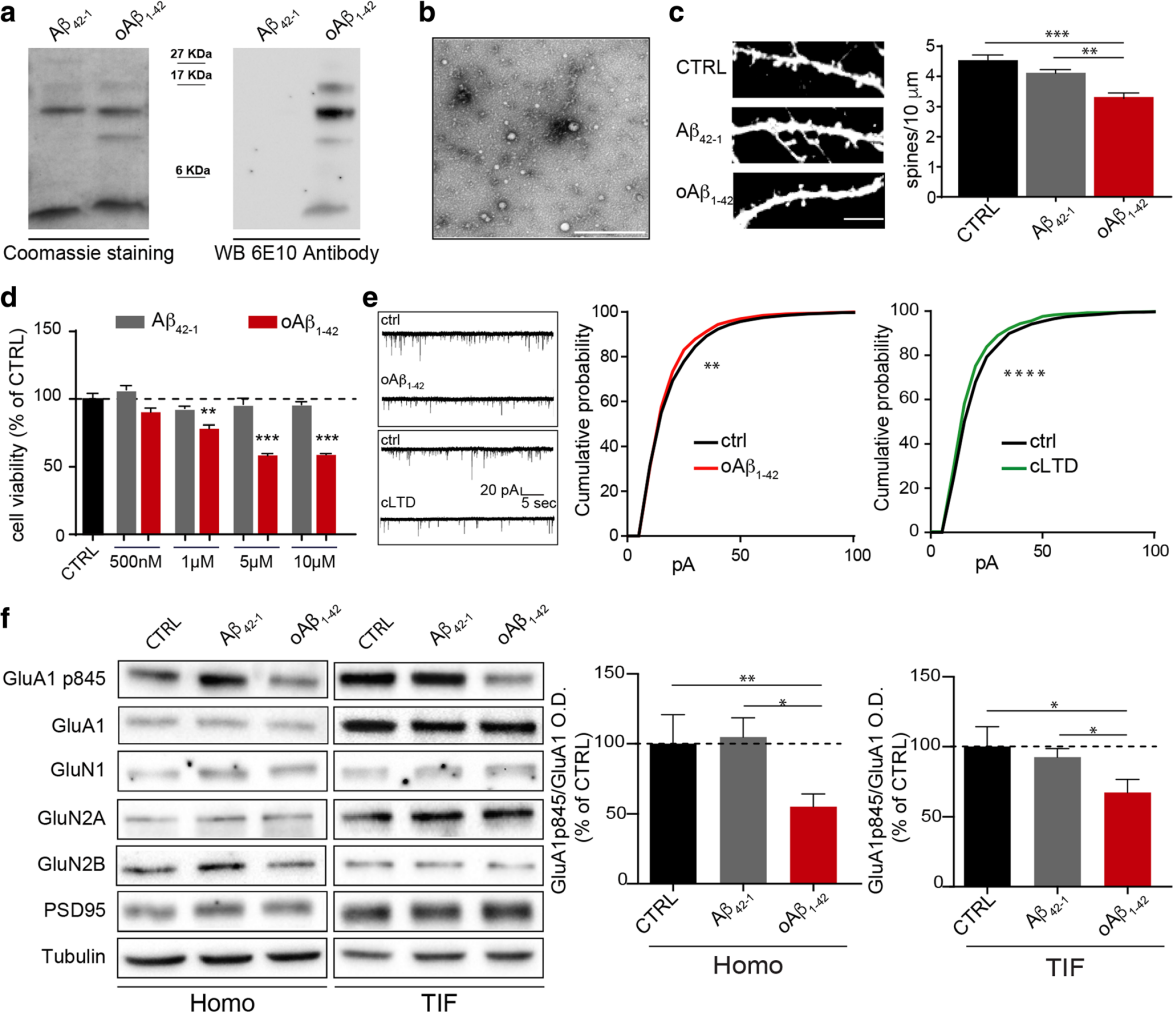
Aβ oligomers characterization and effects on the synapses. **a** A representative Coomassie-stained polyacrylamide gel and Western blot analysis of Aβ_42–1_ and oAβ_1–42_. The image shows the presence of oligomers formation for Aβ_1–42_ and monomer and aggregated forms for Aβ_42–1_. **b** TEM revealed globular but not fibrillar structures for oAβ_1–42_ preparation, scale bar 500 nm. **c** Representative confocal images of GFP-transfected primary hippocampal neurons. The analysis shows that oAβ_1–42_ (500 nM, 24 h) reduces spine density (CTRL 4.58 ± 0.18; Aβ_42–1_ 4.15 ± 0.12; oAβ_1–42_ 3.36 ± 0.18; **p < 0.01, ***p < 0.001; one-way ANOVA, *n* = 34–45). Scale bar 5 μm. **d** MTT test shows that exposure to oAβ_1–42_ for 24 h induces cell death starting from a concentration of 1 μM (**p < 0.01, ***p < 0.001 one-way ANOVA, oAβ_1–42_ vs CTRL, *n* = 4). **e** Representative traces of mEPSCs collected in hippocampal neurons before and after oAβ_1–42_ exposure (upper panel) and before and after chemical LTD (cLTD, lower panel). At least 21 neurons before and after each treatment (oAβ_1–42_ or cLTD) have been analyzed and the related analysis of mEPSC amplitudes, here shown as cumulative probability, includes these *n* of excitatory events: 1550 (before oAβ_1–42_, black line) vs 970 (after oAβ_1–42_, red line), CTRL vs oAβ_1–42_*p* = 0.0075; 2100 (before cLTD, black line) vs 1970 (after cLTD, green line), CTRL vs cLTD *p* < 0.0001. **f** Western blot analysis of synaptic protein levels in total homogenate (HOMO) and synaptic fraction (TIF) upon oAβ_1–42_ treatment (500 nM, 30 min). The quantification shows that oAβ_1–42_ incubation for 30 min induces a decrease of GluA1 phosphorylation at 845-residue (GluA1p845/GluA1, HOMO: CTRL 100 ± 20.76%, Aβ_42–1_ 104.80 ± 13.77%, oAβ_1–42_ 55.22 ± 9.05%; TIF: CTRL 100 ± 14.13%, Aβ_42–1_ 92.40 ± 6.21%, oAβ_1–42_ 67.36 ± 9.30%, *p < 0.05, **p < 0.001, one-way ANOVA, *n* = 7–11). oAβ_1–42_ exposure does not induce significant changes of GluA1, NMDA receptors subunits (GluN1, GluN2A, GluN2B), and PSD-95 expression and synaptic localization

First, oAβ_1–42_ preparation was monitored and controlled by different means. As a negative control, we used a peptide with the reverse sequence of Aβ (Aβ_42–1_). Coomassie staining and Western Blot analysis with an antibody detecting the N-terminus of Aβ (6E10 antibody) indicated that our oAβ_1–42_ preparation resulted in a spectrum of oligomeric Aβ species, from 4 up to 16 kDa (Fig. 1a). Transmission electron microscopy (TEM) analysis of oAβ_1–42_ confirmed the presence of globular, oligomeric structures, while no fibrillar or protofibrillar species were observed (Fig. 1b). Aβ_42–1_ preparation is mainly constituted of monomers and of aggregated species. Western Blot analysis performed with 6E10 antibody showed no signal in Aβ_42–1_ samples, as expected (Fig. 1a).

Consistent with previous studies [14, 15], we found that oAβ_1–42_ species at a concentration of 500 nM applied to hippocampal neurons for 24 hours (h) resulted in a significant reduction in spine density as compared to non-treated cultures (CTRL) or to cells exposed to Aβ_42–1_ (Fig. 1c). Moreover, such concentration of oAβ_1–42_ did not increase the mortality of the cells after 24 h of treatment, different from higher concentrations as 1 μM, 5 μM, and 10 μM (Fig. 1d).

oAβ_1–42_ species have been shown to facilitate synaptic depression of neurons in acute slices [13, 16]. Accordingly, hippocampal cultures exposed to oAβ_1–42_ for 30 min displayed a global synaptic depression as indicated by electrophysiological recordings of excitatory post-synaptic current in miniature (mEPSCs) (Fig. 1e) as well as upon the delivery of the classical LTD protocol (20 μM NMDA and 20 μM glycine for 3 min, chemical LTD; Fig. 1e; [17, 18]). Moreover, we used a biochemical approach and we purified the Triton-insoluble fraction (TIF) that is enriched in post-synaptic proteins. As shown in Fig. 1f, the treatment with oAβ_1–42_ (500 nM, 30 min) induced a significant dephosphorylation of serine 845 of the GluA1 subunit of α-amino-3-hydroxy-5-methyl-4-isoxazolepropionic acid (AMPA) receptors, without any significant change in GluA1 synaptic levels, confirming that this concentration elicits synaptic depression [19]. No changes in the total and synaptic levels of NMDA receptor subunits and in PSD-95 were observed (Fig. 1f; Suppl. Fig. 1A, B).

These results suggest that our oAβ_1–42_ preparation triggers a synaptic depression affecting specifically AMPA mediated conductance.

### oAβ_1–42_ Promote ADAM10 Synaptic Localization Impairing Its Endocytosis

**Fig. 2:**
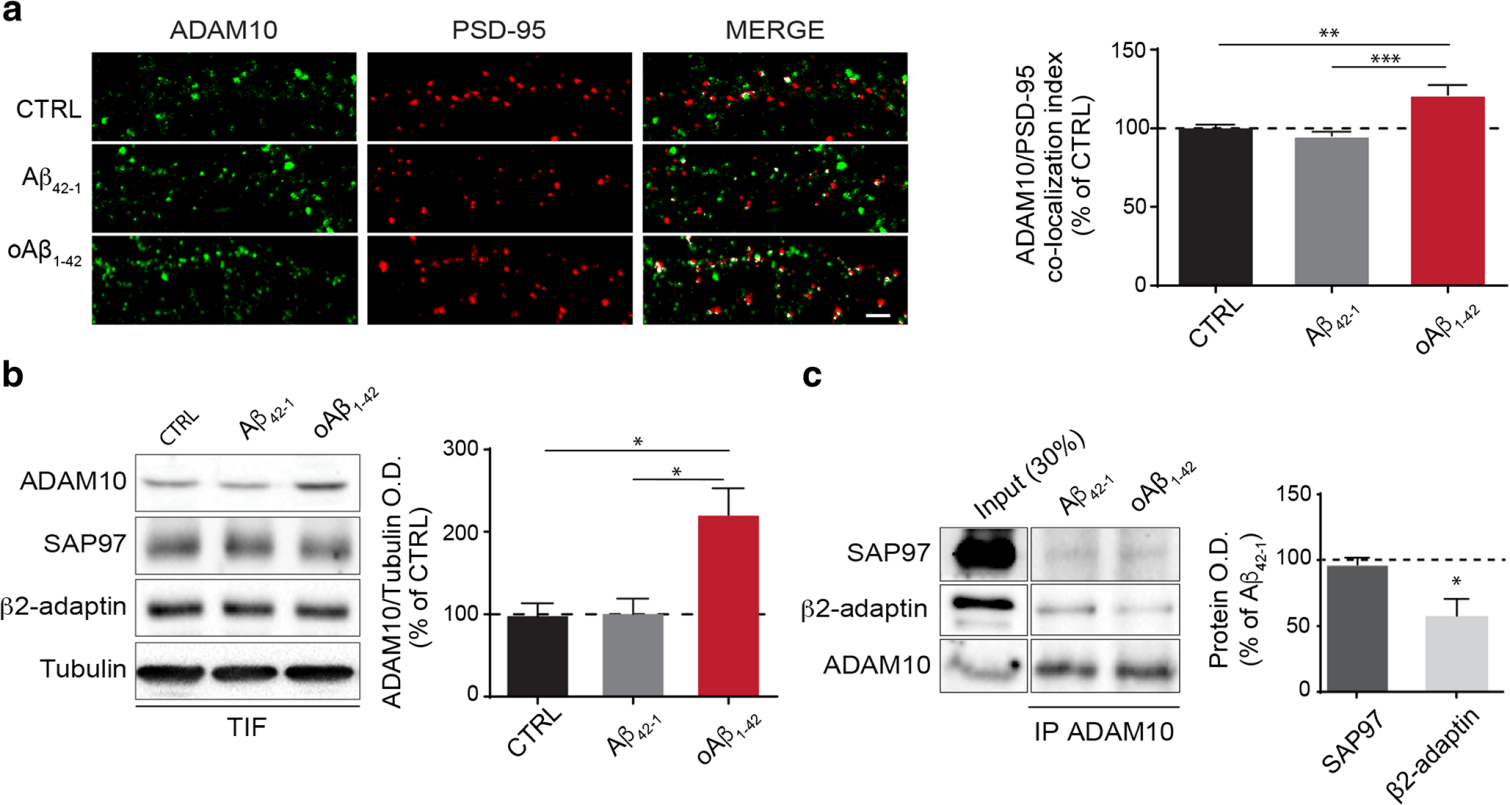
oAβ_1–42_ treatment increases ADAM10 synaptic localization, impairing its endocytosis. **a** Confocal images of primary hippocampal neurons stained with PSD-95 (red) and ADAM10 (green). Cells were untreated (CTRL) or incubated for 30 min with either oAβ_1–42_ or Aβ_42–1_ (500 nM). The challenge with oAβ_1–42_ increases the ADAM10/PSD-95 co-localization index (CTRL 100 ± 2.48%, Aβ_42–1_ 95.59 ± 2.69%, oAβ_1–42_ 123.1 ± 5.86%; *p < 0.05, **p < 0.01 Kruskall-Wallis one-way analysis on variance, *n* = 30). Representative images of ADAM10/PSD-95 co-localization (*white*) are shown on the right, scale bar 5 μm. **b** Representative images of western blot analysis of ADAM10 in the TIF of primary hippocampal neurons. The quantitative analysis shows that oAβ_1–42_ increase ADAM10 synaptic localization (CTRL 100 ± 16.55%, Aβ_42–1_ 102.7 ± 18.9%, oAβ_1–42_ 222 ± 32.46%; *p < 0.05 one-way ANOVA, *n* = 3). **c** Representative images of co-immunoprecipitation between ADAM10 and either SAP97 or β2-adaptin. The quantitative analysis shows that oAβ_1–42_ treatment reduces the interaction with β2-adaptin without affecting the interaction with SAP97 (SAP97 96.95 ± 5.26%, β2-adaptin 57.39 ± 17.81%, *p < 0.05 *t* test oAβ_1–42_ vs Aβ_42–1_, *n* = 3)

Given that ADAM10 synaptic localization is regulated by synaptic plasticity [10], can oAβ_1–42_ modify ADAM10 synaptic levels? To address this issue, we exposed hippocampal neuronal cultures to oAβ_1–42_ (500 nM, 30 min). As shown in Fig. 2a, bath application of oAβ_1–42_ significantly increases the co-localization of ADAM10 with a post-synaptic marker, as PSD-95, along dendrites when compared to untreated or Aβ_42–1_-treated cells.

To further confirm these results by a biochemical approach, we purified the TIF. ADAM10 levels were significantly increased in the TIF upon oAβ_1–42_ treatment (Fig. 2b), indicating that oAβ_1–42_ promote ADAM10 synaptic localization. We have previously shown that LTD induction fosters the SAP97-mediated ADAM10 trafficking to the synapse [10]. However, no alterations of SAP97 synaptic localization were detected upon oAβ_1–42_ treatment (Fig. 2b; Suppl. Fig. 2A). In addition, no modifications of the synaptic levels of β2-adaptin, one of the subunits of the AP2 complex responsible for ADAM10 endocytosis [10], were observed (Fig. 2b; Suppl. Fig. 2B).

To determine the cellular mechanism underlying oAβ_1–42_-induced increase in ADAM10 synaptic localization, we analyzed the association of ADAM10 to SAP97 and AP2 complex. As shown in Fig. 2c, the oAβ_1–42_ treatment does not alter the interaction with SAP97, while significantly decreases the interaction with β2-adaptin, one of the subunits of AP2 complex. These results demonstrate that acute exposure to oAβ_1–42_ affects ADAM10 synaptic localization because of a decrease of endocytosis rather than to an increase of forward trafficking.

### oAβ_1–42_-Triggered ADAM10 Increased Synaptic Localization Is Mediated by the Activation of Synaptic GluN2A-Containing NMDARs

**Fig. 3:**
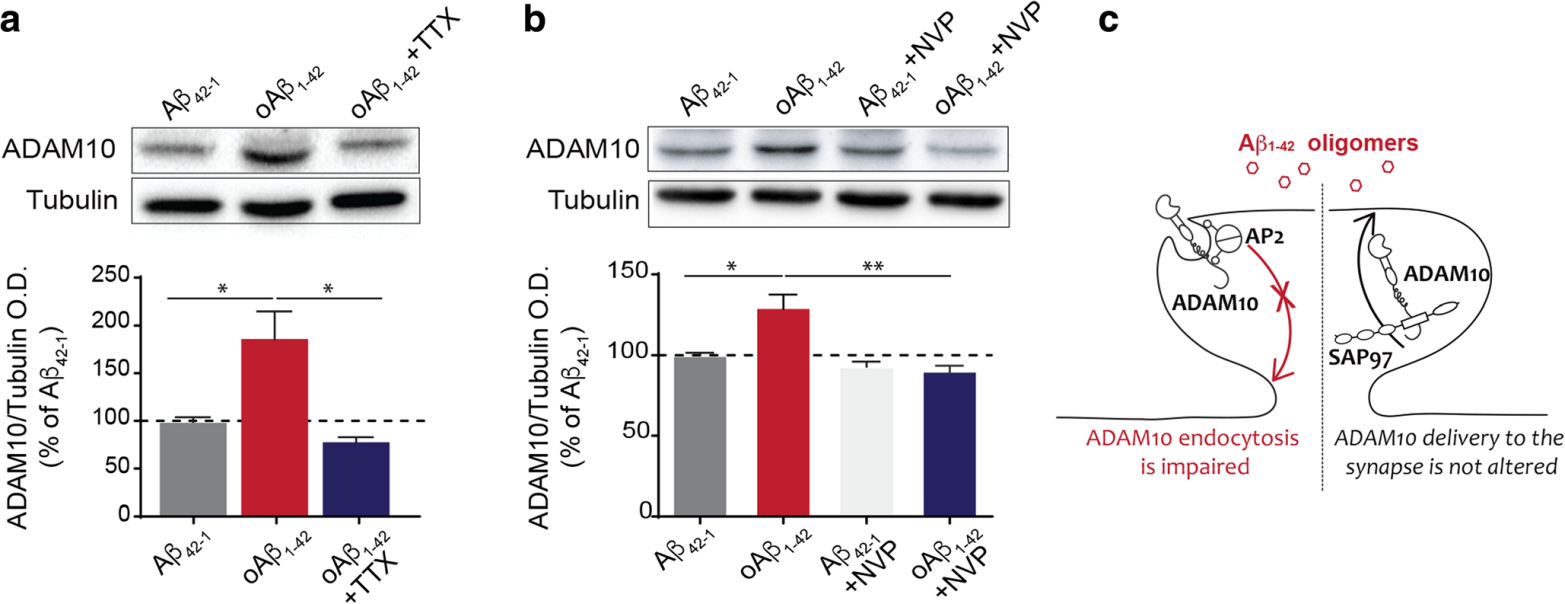
oAβ_1–42_-triggered ADAM10 increase in synaptic localization requires neuronal activity and the activation of GluN2A-containing NMDA receptors. **a** Representative images of Western Blot analysis of ADAM10 levels in the TIF of primary hippocampal neurons treated with TTX for 15 min and then challenged with Aβ_42–1_ or oAβ_1–42_. The quantitative analysis shows that TTX prevents the oAβ_1–42_-induced increase in ADAM10 synaptic localization (Aβ_42–1_ 100 ± 5.80%, oAβ_1–42_ 187.80 ± 29.51%, oAβ_1–42_ + TTX 79.57 ± 5.84%, *p < 0.05, one-way ANOVA, *n* = 3). **b** The presence of NVP-AAM077 (NVP, 50 nM), an inhibitor of GluN2A-containing NMDA receptors, prevents oAβ_1–42_-triggered augment in ADAM10 synaptic levels (Aβ_42–1_ 100 ± 1.52%, oAβ_1–42_ 130 ± 8.91%, Aβ_42–1_ + NVP 92.86 ± 3.19%, oAβ_1–42_ + NVP 90.37 ± 4.41, *p < 0.05, **p < 0.001 one-way ANOVA, *n* = 3). **c** Scheme of the mechanism according to which oAβ_1–42_ affect ADAM10 synaptic localization

To identify which of the cellular pathways triggered by oAβ_1–42_ is responsible for the increase in ADAM10 synaptic localization, we analyzed the effect of the blockade of different events. First, we took advantage of the action potential blocker tetrodotoxin (TTX) (500 nM), and we observed that TTX pre-incubation prevents the oAβ_1–42_-induced increase of ADAM10 in the TIF (Fig. 3a), indicating the involvement of neuronal synaptic activity.

In the adult forebrain, synaptic NMDA receptors are predominantly di-heteromeric GluN1/GluN2A and tri-heteromeric GluN1/GluN2A/GluN2B receptors [20, 21]. In light of this observation and considering the key role of GluN2A-containing NMDA receptors in plasticity phenomena [22, 23], we examined the effect of NVP-AAM077, a GluN2A-preferring antagonist [24]. The analysis of ADAM10 levels in the TIF shows that the presence of NVP-AAM077 fully prevents the increased localization of ADAM10 in the synapses induced by oAβ_1–42_ (Fig. 3b). Notably, the presence of ifenprodil, an antagonist of GluN2B-containing NMDA receptors, does not affect the oAβ_1–42_-triggered increase in ADAM10 synaptic levels, thus indicating the specific involvement of GluN2A-containing NMDA receptors (Suppl. Fig. 3).

## Discussion

In this study, we provide evidence for aberrant plasticity phenomena by which oAβ_1–42_ control synaptic function and the generation of Aβ_1–42_ itself. Primary hippocampal cultures were treated with a preparation of oAβ_1–42_ able to induce synaptic depression in 30 min and spine loss in 24 h, in the absence of cell death. In these experimental conditions, oAβ_1–42_ short-term exposure results in an increase in ADAM10 synaptic availability. Although it has been shown that LTD fosters SAP97-mediated ADAM10 delivery to the post-synaptic compartment [10], oAβ_1–42_ treatment leads to a decrease in the association between ADAM10 and AP2 complex, which is responsible for the endocytosis of the enzyme, without affecting the binding to SAP97 (Fig. 3c). Therefore, the increase in ADAM10 synaptic localization is due to the impairment of its endocytosis rather than to a stimulation of its forward trafficking, suggesting that the molecular pathways underlying physiological LTD and regulating ADAM10 are profoundly different from those responsible for oAβ_1–42_-induced depression.

Considering that several studies demonstrated that oAβ_1–42_ inhibit the maintenance of hippocampal LTP [8, 25, 26] and that ADAM10 endocytosis is regulated by LTP, we can also hypothesize that the oAβ_1–42_-induced impairment in ADAM10 endocytosis is in line with a disorder in mechanism of LTP. Thus, overall our data indicate that oAβ_1–42_ triggers aberrant plasticity phenomena.

We provide also a mechanistic inside for this aberrant plasticity of oAβ_1–42_ action. oAβ_1–42_ engage a pathway that requires neuronal activity and the activation of the GluN2A-containing NMDA receptors. Even if several studies reported the role of the extra-synaptic GluN2B-containing NMDA receptors [27–29], our data highlight that acute exposure to oAβ_1–42_ triggers a synaptic event that involves GluN2A-containing NMDA receptors. Indeed, the presence of an inhibitor of GluN2B-containing NMDA receptors does not prevent the oAβ_1–42_-induced increase in ADAM10 synaptic localization.

Taken together, these data suggest that a short-term exposure to oAβ_1–42_ engages a negative feedback mechanism according to which oAβ_1–42_ can downregulate Aβ generation through the modulation of ADAM10 synaptic availability. Moreover, the increased activity of ADAM10 towards its synaptic substrates could tune synaptic transmission and structural plasticity. It has been shown that the sAPPα, which is released after ADAM10 cleavage of APP, is able to acutely modulate synaptic strength when applied in vitro [30]. Furthermore, ADAM10-mediated shedding of N-cadherin controls spine shaping and AMPA receptors function [31].

Overall, here, we show that oAβ_1–42_ can trigger aberrant plasticity pathways and, thereby, affect synaptic plasticity. Since the synapses are considered to be an early site of pathology in AD [32] and loss of synapses is the best pathologic correlate of cognitive impairment in AD patients [33], understanding the molecular underpinnings leading to synaptic dysfunction will aid in the development of tailored synapse-targeted therapies for AD.

## Methods

### Amyloid Oligomers Preparation

Aβ_1–42_ and Aβ_42–1_ peptides were purchased from Bachem (Bubendorf, Switzerland) and oligomers were prepared according to [34]. The lyophilized peptides were dissolved in 1,1,1,3,3,3-hexafluoro-2-propanol (HFIP; Sigma, St. Louis, MO, USA) and aliquoted before removing HFIP. oAβ_1–42_ were obtained by incubating at 4 °C for 24 h in Neurobasal medium without Phenol red. The quality of the oligomer preparation was controlled separating the protein onto a 13% Tris-Tricine gels and performing Coomassie staining and western blots against the amyloid-β peptide (6E10; Covance, CA, USA). To analyze the presence of oligomeric and fibrillar forms, TEM experiments were performed by applying 5 μl of protein suspension to a glow-discharge coated carbon grid (Cu 300 mesh, Electron Microscopy Sciences, PA, USA) for 1 min and then negatively stained with 2% Uranyl acetate. Sample was observed at an EFTEM Leo912ab (Zeiss, Germany) operating at 100 kV and digital images were acquired by a CCD camera 1kx1k (Proscan, Germany) and iTEM software (Olympus, Germany).

### Neuronal Cultures Preparation, Transfection, and Treatments

Primary hippocampal neurons cultures were prepared from embryonic day 18–19 rat hippocampi as previously described [35]. The Institutional Animal Care and Use Committee of University of Milan and the Italian Ministry of Health (#326/2015) approved all the experiments involving primary neuronal cultures preparation.

Neurons were transfected with eGFP plasmid using the calcium phosphate precipitation method at 10 days in vitro (*DIV*) for spines density analysis. All the treatments were performed at *DIV14* using the following reagents concentrations: either oAβ_1–42_ or Aβ_42–1_ 500 nM (30 min), NVP-AAM077 (GluN2A-containing NMDA receptor antagonist, TOCRIS, Bristol, UK) 50 nM (pre-incubation of 15 min), TTX (Tetrodotoxin, TOCRIS) 500 nM (pre-incubation of 15 min), and ifenprodil (GluN2B-containing NMDA receptor antagonist, TOCRIS) 3 μM (pre-incubation of 5 min). Neuronal cultures were treated with either oAβ_1–42_ or Aβ_42–1_ at different concentrations (500 nM, 1 μM, 5 μM, and 10 μM) and, after 24 h, the MTT test was performed according to [36] to evaluate the cells viability.

### Synaptic Fraction Purification, Western Blot, and Co-immunoprecipitation Analysis

After treatment, samples were processed for the purification of the Triton-insoluble fraction (TIF), a fraction enriched in post-synaptic density proteins [35]*.* After quantification, total homogenate and TIF proteins were resolved with SDS-PAGE method; co-immunoprecipitation experiments were performed as described in [10, 35].

### Antibodies

The following antibodies (Ab) were used: ADAM10 purchased from Abcam ab39153 (Cambridge, UK), SAP97 from Stressgen ADI-VAM-PS005-D (San Diego, CA, USA), β2-Adaptin from BD Bioscience 610382 (NJ, USA), Tubulin T9026 and GluN2A M264 from Sigma-Aldrich, GluA1 75-327, PSD-95 75-028, GluN2B 75-097, and GFP 75-132 from Neuromab (Davis, CA, USA), GluA1-p845 04-1073 and 6E10 SIG39320-200 (Covance) from Millipore (Billenca, MA, USA), and GluN1 320500 from Thermo Fisher (Waltham, MA, USA). Peroxidase-conjugated secondary Abs were purchased from Bio-Rad (Hercules, CA, USA). AlexaFluor secondary Abs were purchased from Thermo Fisher.

### Immunocytochemistry and Confocal Microscope Acquisition

For ADAM10/PSD-95 co-localization and spine morphology studies, treated hippocampal neurons were fixed 7 min in 4% paraformaldehyde plus 4% sucrose in phosphate buffered saline (PBS) at room temperature. Then, cells were extensively washed with PBS supplemented with CaCl_2_ and MgCl_2_, permeabilized with 0.2% Triton-X100 and incubated for 2 h at room temperature with 5% BSA in PBS. Primary and secondary antibodies were applied in 5% bovine serum albumin (BSA) in PBS. Cells were labeled with primary antibodies overnight at 4 °C. Cells were washed and then incubated with secondary antibodies for 1 h at room temperature. Cells were then washed in PBS and mounted on glass slides with Fluoromount mounting medium (Sigma-Aldrich). Fluorescence images were acquired by using Zeiss Confocal LSM510 system (Zeiss, Jena, Germany) with a sequential acquisition setting at 1024 × 1024 pixels resolution; for each image, two up to four 0.5-μm sections were acquired and a z projection was obtained [31].

### Cell Culture Electrophysiology

Whole-cell patch-clamp recordings of mEPSCs were obtained from DIV 15–16 neurons using a Multiclamp700A amplifier (Molecular Devices) and pClamp-10 software (Axon Instruments, Foster City, CA). Recordings were performed in the voltage-clamp mode. Currents were sampled at 5 kHz and filtered at 2–5 kHz. Recording pipettes, tip resistances of 3–5 MΩ were filled with the intracellular solution of the following composition (in mM): 130 potassium gluconate, 10 KCl, 1 EGTA, 10 Hepes, 2 MgCl2, 4 MgATP, 0.3 Tris-GTP. At the beginning of the experiment, mEPSCs have been recorded in the external solution [Krebs’ Ringer’s-HEPES (KRH)] with the following composition (in mM): 125 NaCl, 5 KCl, 1.2 MgSO4, 1.2 KH2PO4, 2 CaCl2, 6 glucose, 25 HEPES-NaOH, pH 7.4 in which also TTX (0.5 μM), bicuculline (20 μM, Tocris, Bristol, UK), and strychnine (1 μM, Sigma-Aldrich, Milan, Italy) were included. Then, to induce chemical LTD, we applied NMDA (20 μM) and glycine (20 μM, Sigma-Aldrich, Milan, Italy) for 3 min at room temperature in Mg^2+^-free KRH containing TTX (0.5 μM), bicuculline (20 μM), and strychnine (1 μM). Thirty minutes after this treatment, mEPSCs have been collected again in the starting KRH solution. Synaptic depression has been also induced in cultures by 30 min of oAβ_1–42_ (500 nM) in Mg^2+^-free KRH containing only TTX (0.5 μM), bicuculline (20 μM) and strychnine; at the end of this treatment, mEPSCs have been recorded in normal KRH and analyzed. Off-line analysis of miniature events was performed by the use of Clampfit- pClamp-10 software.

### Data Quantification and Statistical Analysis

Quantification of Western Blot analysis was performed by means of computer-assisted imaging (Image Lab, Biorad). The levels of the proteins were expressed as relative optical density (OD) measurements and normalized on tubulin. Values are expressed as mean ± S.E.M. of at least three independent experiments.

Co-localization analysis was performed using Zeiss AIM 4.2 software and spines analysis was performed with ImageJ software (National Institute of Health, Bethesda, MD, USA). For co-localization, and morphological analysis, cells were chosen randomly for quantification from 4 different coverslips (2–3 independent experiments), images were acquired using the same settings/exposure times, and at least 10 cells for each condition were analyzed. Statistical evaluations were performed by using 2-tailed Student’s *t* test (a *p* value less than 0.05 was considered significant) or, when appropriate, by using one-way ANOVA followed by Bonferroni’s post hoc test or Kruskal-Wallis analysis of variance followed by Dunn’s post hoc test.

## Electronic supplementary material

Suppl. Fig. 1 oAβ_1–42_ short exposure does not affect the expression and synaptic levels of GluN1, GluN2A, GluN2B and PSD-95. **A)** Quantitative analysis of Western Blot analysis of homogenate reported in Fig.1 F (GluN1, CTRL 100 ± 33.44%, Aβ _42–1_ 70.59 ± 17.76%, oAβ_1–42_ 109.3 ± 14.55%; GluN2A, CTRL 100 ± 24.25, Aβ _42–1_ 119.9 ± 34.25%, oAβ_1–42_ 80.28 ± 8.62%; GluN2B, CTRL 100 ± 30.65%, Aβ _42–1_ 90.94 ± 16.17%, oAβ_1–42_ 99.64 ± 45.05%; PSD-95, CTRL 100 ± 20.64%, Aβ _42–1_ 132.2 ± 6.41%, oAβ_1–42_ 111 ± 28.41%; *p* > 0.05, one-way ANOVA, *n* = 5); **B)** Quantitative analysis of Western Blot analysis of TIF reported in Fig.1 F (GluN1, CTRL 100 ± 26.01%, Aβ _42–1_ = 83.29 ± 32.03%, oAβ_1–42_ 72.32 ± 13.9%; GluN2A CTRL 100 ± 16.60%, Aβ_42–1_ 106.9 ± 10.78%, oAβ_1–42_ 104.9 ± 18.36%; GluN2B, CTRL 100 ± 25.43%, Aβ_42–1_ 117.3 ± 19.03, oAβ_1–42_ 102.0 ± 14.97%; PSD-95, CTRL 100 ± 8.03, Aβ_42–1_ 106.2 ± 5.13, oAβ_1–42_ 102 ± 5.84; *p* > 0.05, one-way ANOVA, n = 5). (PNG 43 kb)

High Resolution Image (TIF 130 kb)

Suppl. Fig. 2 oAβ_1–42_ short exposure does not affect the synaptic localization of SAP97 and β2-adaptin. **A)** Quantitative analysis of Western Blot analysis of SAP97 levels in TIF reported in Fig. 2B (CTRL 100 ± 37.86%, Aβ _42–1_ 123.6 ± 29.07%, oAβ_1–42_ 96.75 ± 21.64%; *p* > 0.05, one-way ANOVA, n = 5); **B)** Quantitative analysis of Western Blot analysis of β2-adaptin in TIF reported in Fig.2B (CTRL 100 ± 6.96%, Aβ _42–1_ = 101.50 ± 9.20%, oAβ_1–42_ 110.60 ± 8.35%; *p* > 0.05, one-way ANOVA, n = 5) (PNG 44 kb)

High Resolution Image (TIF 109 kb)

Suppl. Fig. 3 oAβ_1–42_-induced increase in ADAM10 synaptic availability does not involve the activation of GluN2B-containing NMDA receptors. The presence of ifenprodil (IF, 3 μM), an inhibitor of GluN2B-containing NMDA receptors, does not affect oAβ_1–42_-triggered augment in ADAM10 synaptic levels (Aβ _42–1_ 100 ± 33.43%, oAβ_1–42_ = 230.50 ± 68.24%, IF + oAβ_1–42_ 158.30 ± 29.30%; * *p* < 0.05, one-way ANOVA, *n* = 7) (PNG 66 kb)

High Resolution Image (TIF 134 kb)

## Abbreviations

AD: Alzheimer’s disease
ADAM10: A disintegrin and metalloproteinase 10
AMPA receptors: α-amino-3-hydroxy-5-methyl-4-isoxazolepropionic acid receptor
Aβ_1–42_: amyloid-β_1–42_
APP: amyloid-β precursor protein
Ab: antibody
BSA: bovine serum albumin
CTRL: non-treated cultures
DIV: days in vitro
LTD: long-term depression
LTP: long-term potentiation
mEPSCs: excitatory post-synaptic current in miniature
NMDA receptors: N-methyl-D-aspartate (NMDA) receptors
oAβ_1–42_: oligomers of Aβ_1–42_
PBS: phosphate buffered saline
TEM: transmission electron microscopy
TIF: Triton-insoluble fraction
TTX: tetrodotoxin
