# Supplementary figures and images for: Amyloid-β Oligomers Regulate ADAM10 Synaptic Localization Through Aberrant Plasticity Phenomena
Source: Mol Neurobiol. 2019 Apr 13;56(10):7136–43. doi: 10.1007/s12035-019-1583-5 (PMC6728288; doi:10.1007/s12035-019-1583-5)

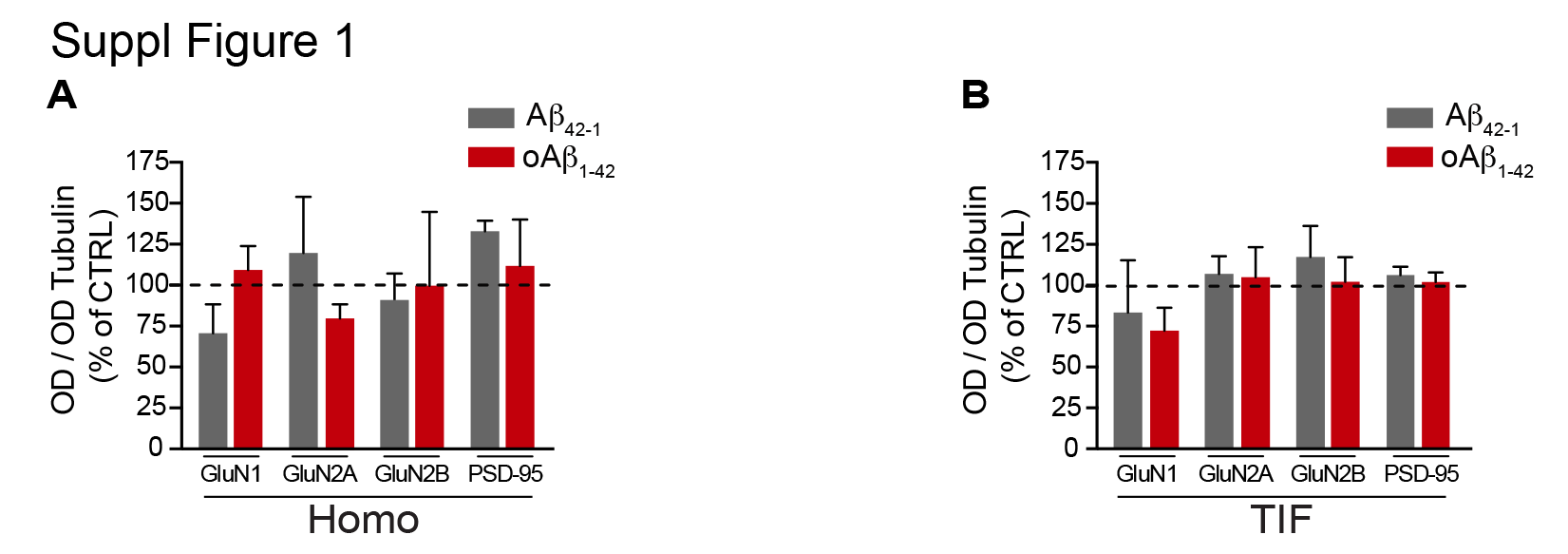

Supplement: Supplementary file 1 — oAβ1–42 short exposure does not affect the expression and synaptic levels of GluN1, GluN2A, GluN2B and PSD-95. A) Quantitative analysis of Western Blot analysis of homogenate reported in Fig.1 F (GluN1, CTRL 100 ± 33.44%, Aβ 42–1 70.59 ± 17.76%, oAβ1–42 109.3 ± 14.55%; GluN2A, CTRL 100 ± 24.25, Aβ 42–1 119.9 ± 34.25%, oAβ1–42 80.28 ± 8.62%; GluN2B, CTRL 100 ± 30.65%, Aβ 42–1 90.94 ± 16.17%, oAβ1–42 99.64 ± 45.05%; PSD-95, CTRL 100 ± 20.64%, Aβ 42–1 132.2 ± 6.41%, oAβ1–42 111 ± 28.41%; p > 0.05, one-way ANOVA, n = 5); B) Quantitative analysis of Western Blot analysis of TIF reported in Fig.1 F (GluN1, CTRL 100 ± 26.01%, Aβ 42–1 = 83.29 ± 32.03%, oAβ1–42 72.32 ± 13.9%; GluN2A CTRL 100 ± 16.60%, Aβ42–1 106.9 ± 10.78%, oAβ1–42 104.9 ± 18.36%; GluN2B, CTRL 100 ± 25.43%, Aβ42–1 117.3 ± 19.03, oAβ1–42 102.0 ± 14.97%; PSD-95, CTRL 100 ± 8.03, Aβ42–1 106.2 ± 5.13, oAβ1–42 102 ± 5.84; p > 0.05, one-way ANOVA, n = 5). (PNG 43 kb) [file 12035_2019_1583_Fig4_ESM.png]

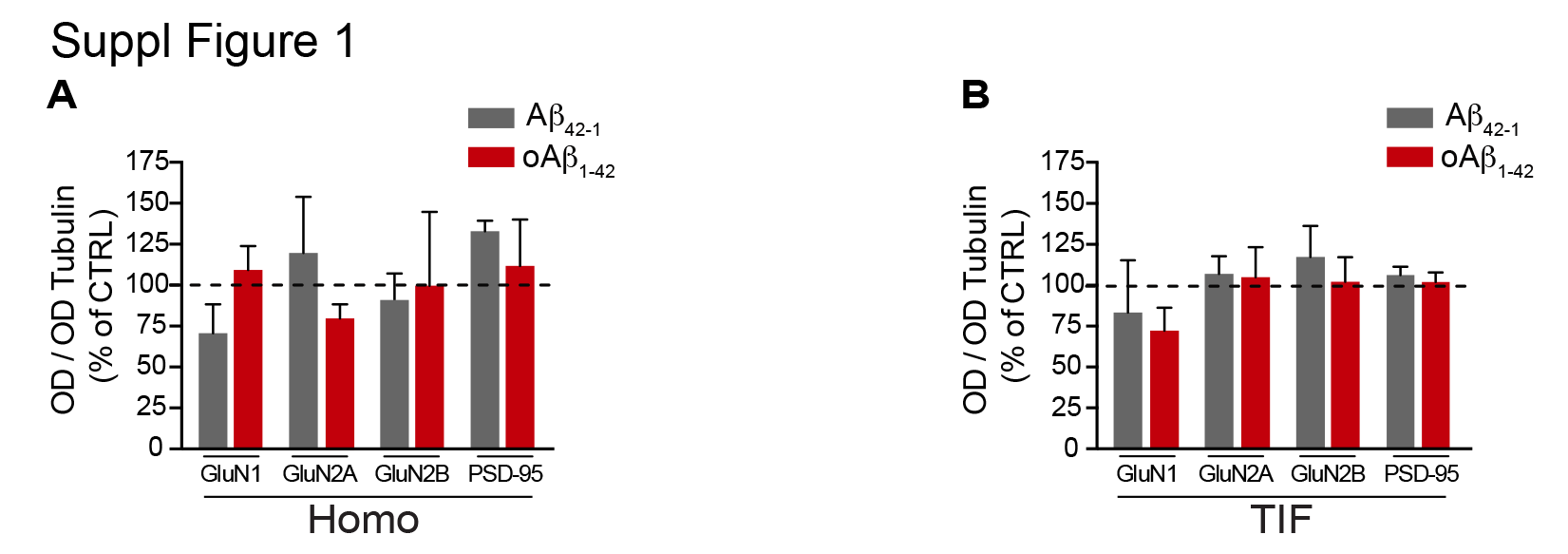

Supplement: Supplementary file 2 — High Resolution Image (TIF 130 kb) [file 12035_2019_1583_MOESM1_ESM.tif]

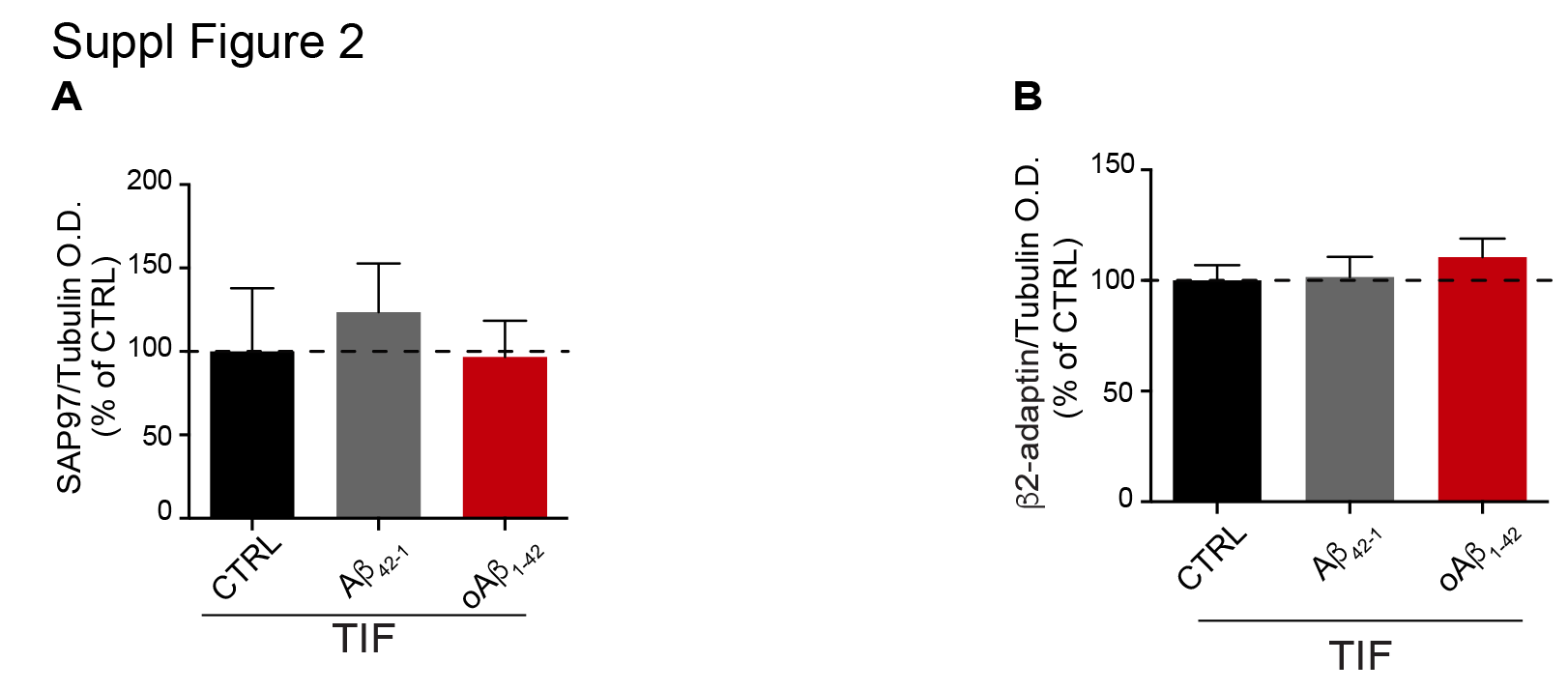

Supplement: Supplementary file 3 — oAβ1–42 short exposure does not affect the synaptic localization of SAP97 and β2-adaptin. A) Quantitative analysis of Western Blot analysis of SAP97 levels in TIF reported in Fig. 2B (CTRL 100 ± 37.86%, Aβ 42–1 123.6 ± 29.07%, oAβ1–42 96.75 ± 21.64%; p > 0.05, one-way ANOVA, n = 5); B) Quantitative analysis of Western Blot analysis of β2-adaptin in TIF reported in Fig.2B (CTRL 100 ± 6.96%, Aβ 42–1 = 101.50 ± 9.20%, oAβ1–42 110.60 ± 8.35%; p > 0.05, one-way ANOVA, n = 5) (PNG 44 kb) [file 12035_2019_1583_Fig5_ESM.png]

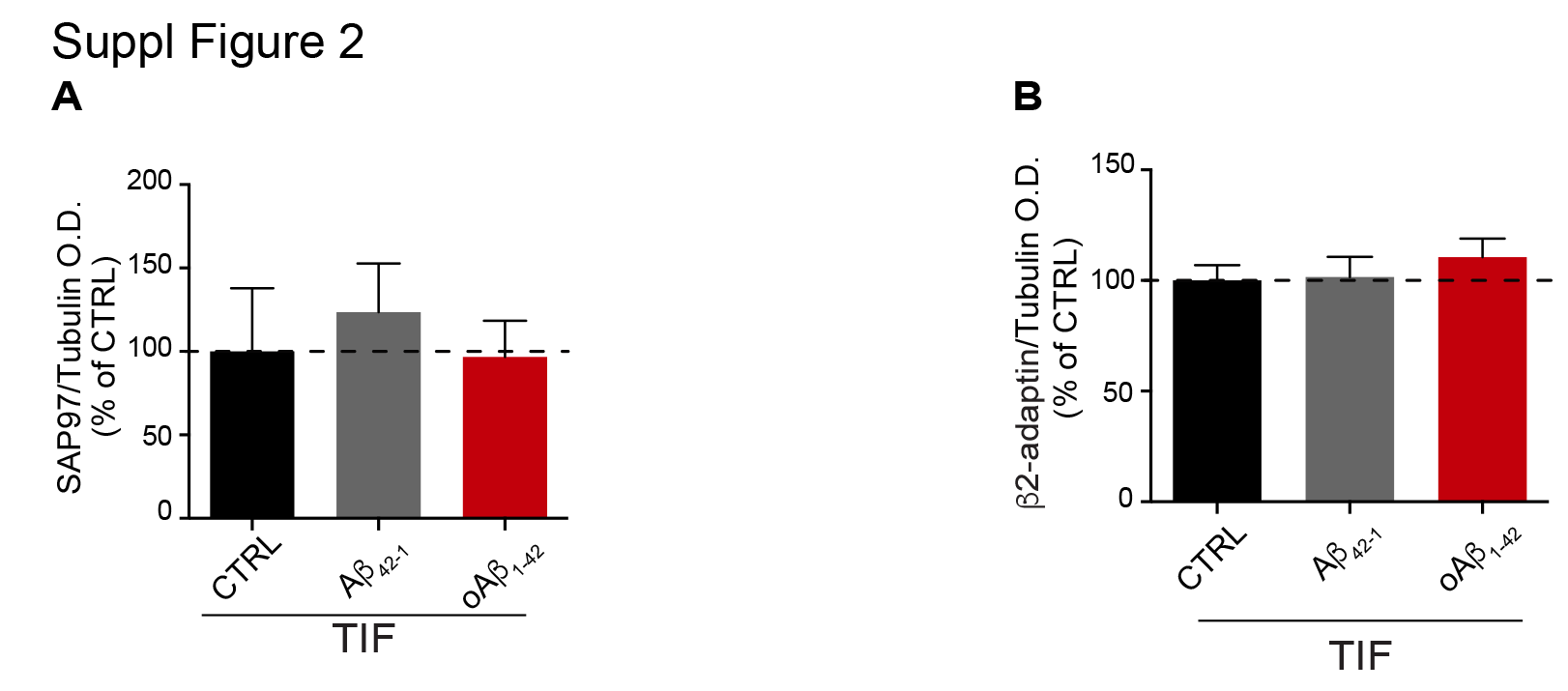

Supplement: Supplementary file 4 — High Resolution Image (TIF 109 kb) [file 12035_2019_1583_MOESM2_ESM.tif]

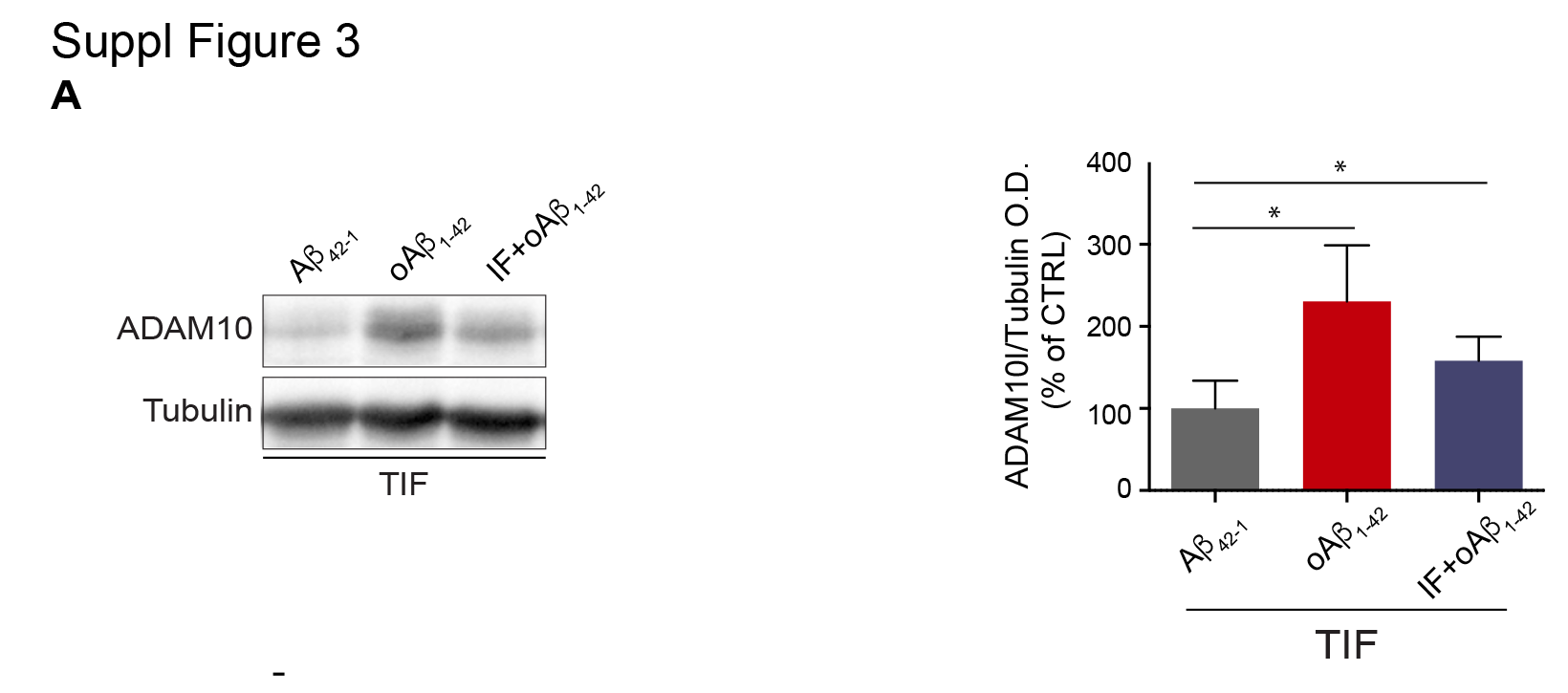

Supplement: Supplementary file 5 — oAβ1–42-induced increase in ADAM10 synaptic availability does not involve the activation of GluN2B-containing NMDA receptors. The presence of ifenprodil (IF, 3 μM), an inhibitor of GluN2B-containing NMDA receptors, does not affect oAβ1–42-triggered augment in ADAM10 synaptic levels (Aβ 42–1 100 ± 33.43%, oAβ1–42 = 230.50 ± 68.24%, IF + oAβ1–42 158.30 ± 29.30%; * p < 0.05, one-way ANOVA, n = 7) (PNG 66 kb) [file 12035_2019_1583_Fig6_ESM.png]

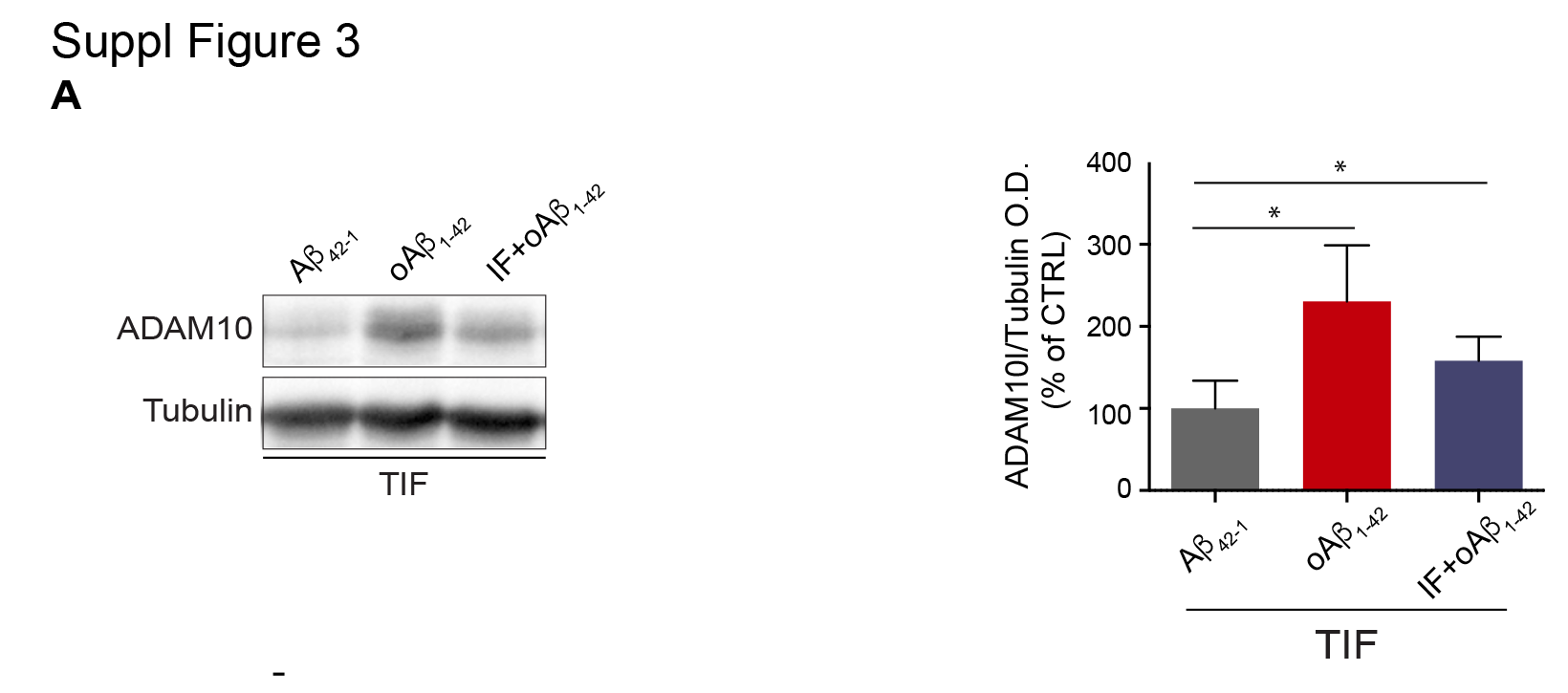

Supplement: Supplementary file 6 — High Resolution Image (TIF 134 kb) [file 12035_2019_1583_MOESM3_ESM.tif]
